# Comparative Botulinum Toxin Injection Strategies Beyond the Masseter for Bruxism: A Scoping Review

**DOI:** 10.3390/jcm15197712

**Published:** 2026-10-06

**Authors:** Doungkamol Siri-Archawawat, Arunrat Dumkliang, Chime Eden, Weeratian Tawanwongsri

**Affiliations:** 1 Division of Neurology, Department of Internal Medicine, School of Medicine, Walailak University, Nakhon Si Thammarat 80160, Thailand; 2 Department of Otorhinolaryngology, Thung Song Hospital, Nakhon Si Thammarat 80110, Thailand; 3 Department of Dermatology, Jigme Dorji Wangchuck National Referral Hospital, Thimphu 11001, Bhutan; 4 Division of Dermatology, Department of Internal Medicine, School of Medicine, Walailak University, Nakhon Si Thammarat 80160, Thailand

**Keywords:** bruxism, botulinum toxin type A, masseter muscle, masticatory muscles, temporal muscle, injection strategy

## Abstract

**Background:** Botulinum toxin type A is used to reduce excessive masticatory muscle activity in bruxism, but whether extending injection beyond the masseter provides additional benefit remains uncertain. This scoping review aimed to map comparative evidence on expanded botulinum toxin muscle targeting for bruxism and assess whether available studies provide evidence of incremental clinical benefit beyond masseter-only injection. **Methods:** PubMed, Scopus, the Cochrane Central Register of Controlled Trials, and the World Health Organization International Clinical Trials Registry Platform were searched from inception to 3 August 2026. Eligible studies directly compared botulinum toxin injection strategies differing in the muscles targeted. Data were charted and synthesized descriptively. The protocol was prospectively registered with INPLASY (INPLASY202680008). **Results:** Five small randomized trials involving 133 participants were included, of whom 116 contributed to the comparative analyses. Expanded strategies added the temporalis, anterior digastric, lateral pterygoid, or medial pterygoid. Additional clinical benefit of broader targeting was not clearly demonstrated, although some studies reported greater reductions in activity of the additionally injected muscle. In all studies, the masseter dose was held constant while additional muscles were injected, resulting in higher total toxin doses and preventing separation of anatomical targeting effects from dose escalation. Safety evidence was insufficient because reporting was limited and heterogeneous, and the small overall sample precluded adequate characterization of the safety profile. **Conclusions:** The available evidence is preliminary and insufficient to determine whether routinely extending botulinum toxin injection beyond the masseter provides additional clinical benefit. Future adequately powered, dose-controlled trials should prioritize patient-important outcomes and distinguish the effects of anatomical targeting from total toxin dose.

## 1. Introduction

Bruxism is repetitive masticatory muscle activity involving clenching or grinding of the teeth and bracing or thrusting of the mandible, with distinct sleep and awake manifestations [1]. Current consensus considers bruxism a motor behavior rather than inherently a disorder [1,2]. When clinically consequential, it may contribute to tooth wear, masticatory muscle pain, temporomandibular disorders, and impaired oral function [3,4,5,6].

Bruxism involves the coordinated activity of several masticatory muscles. The masseter and temporalis are principal jaw-closing muscles, whereas the pterygoid and suprahyoid muscles contribute to mandibular protrusion, lateral excursion, opening, and stabilization [7,8,9]. This offers an anatomical justification for the expansion of botulinum toxin treatment beyond the masseter. However, injection strategies vary in muscle selection, toxin preparation, dose, injection points, and guidance technique, and direct comparative evidence for additional muscle targeting remains limited [10,11,12,13,14,15,16,17]. Previous systematic reviews have primarily examined the overall efficacy and safety of botulinum toxin for bruxism rather than the incremental value of extending treatment beyond the masseter [10,12,16,18]. Thus, whether adding other masticatory muscles provides additional clinical benefit compared with masseter-only injection remains unclear. Accordingly, this scoping review aimed to map the comparative evidence on expanded botulinum toxin muscle targeting for bruxism and assess whether available studies indicate incremental clinical benefit beyond masseter-only injection, while characterizing injection protocols, physiological outcomes, safety findings, and remaining evidence gaps.

## 2. Materials and Methods

### 2.1. Protocol and Reporting Framework

This scoping review was conducted in accordance with the JBI methodology for scoping reviews and is reported in accordance with the Preferred Reporting Items for Systematic Reviews and Meta-Analyses extension for Scoping Reviews (PRISMA-ScR) [19]. The completed PRISMA-ScR checklist is provided as Supplementary Table S1 [20]. The protocol was registered prospectively with the International Platform of Registered Systematic Review and Meta-analysis Protocols (INPLASY) on 2 August 2026 (INPLASY202680008; doi: 10.37766/inplasy2026.8.0008).

A scoping review was chosen to map the available comparative evidence and identify evidence gaps rather than estimate a pooled treatment effect. Using the Population–Concept–Context (PCC) framework [21], the population comprised children or adults with sleep, awake, mixed, or unspecified bruxism; the concept was comparative botulinum toxin injection strategies differing in the muscles targeted; and the context was any clinical or research setting. The review question was, “What comparative evidence exists on botulinum toxin injection strategies extending beyond the masseter, and does it indicate additional clinical benefit over masseter-only injection in individuals with bruxism?”

### 2.2. Eligibility Criteria

Eligible participants were children or adults with sleep, awake, mixed, or unspecified bruxism, whether primary or secondary. Both pediatric and adult populations were eligible to capture the full extent of comparative evidence; participant age was charted, and pediatric and adult evidence was to be synthesized separately because of potential differences in anatomy, dosing, and clinical outcomes. Studies were classified according to the bruxism phenotype reported by the original investigators as sleep, awake, mixed, or unspecified bruxism, together with the assessment method used. Assessment approaches were categorized as instrumental, including electromyography or polysomnography, or non-instrumental, including self-report, questionnaire, and/or clinical examination, consistent with current consensus frameworks for bruxism assessment [1,22]. These phenotypes and assessment approaches were not considered diagnostically equivalent and were not pooled or directly compared across studies. Instead, they were retained as sources of clinical heterogeneity and considered explicitly in the narrative synthesis. Participants were not retrospectively reclassified beyond the information reported in the original studies. Studies of populations with comorbid conditions such as temporomandibular disorders, myofascial pain, or neurological disease were included only when bruxism was explicitly identified or when data for participants with bruxism could be extracted separately. The concept of interest was the comparative use of botulinum toxin injection strategies that differ in targeted muscles, namely masseter-only injection versus injection of the masseter plus one or more additional muscles such as the temporalis, medial or lateral pterygoid, digastric, suprahyoid, or other muscles directly involved in mandibular movement or stabilization. Comparisons were eligible when the study groups differed in the anatomical distribution of botulinum toxin injection; the predefined classification rules used for synthesis are described in Section 2.7. No restrictions were applied to the toxin formulation, injection guidance method, dose, number of injection points, or follow-up duration. Studies conducted in any clinical or research setting and country were considered.

Randomized parallel-group trials, randomized crossover trials, and prospective non-randomized comparative interventional studies were eligible. Eligibility was restricted to reports published in English because resources for reliable translation were unavailable. The restriction was applied during screening, except in Scopus, where an English-language filter was applied at the search stage. Trial registrations, protocols, and conference abstracts meeting the same criteria were retained and linked to related publications. Records without results were mapped descriptively without contributing to conclusions on effectiveness or safety. Studies were excluded if they compared botulinum toxin only with a placebo, no treatment, or another intervention without contrasting muscle-targeting strategies, such as single-arm studies, all retrospective designs (whether comparative or non-comparative), case reports and case series, reviews and editorials, animal and laboratory studies, cosmetic masseter reduction studies without bruxism, and studies of temporomandibular disorders or facial pain without an identifiable bruxism population. Eligible outcomes included bruxism frequency or intensity, electromyographic or polysomnographic measures, pain, bite force, jaw function, muscle activity or thickness, patient-reported improvement, satisfaction, quality of life, duration of effect, and adverse events. Studies reporting only one relevant outcome were retained.

### 2.3. Information Sources and Search Strategy

PubMed/MEDLINE, Scopus, the Cochrane Central Register of Controlled Trials (CENTRAL), and the WHO International Clinical Trials Registry Platform (ICTRP) were searched from inception to 3 August 2026. These sources were selected to provide complementary coverage of biomedical literature, multidisciplinary indexing, controlled trials, and registered trials. The strategy combined controlled vocabulary and free-text terms for bruxism, botulinum toxin, and the relevant masticatory muscles, and was developed in PubMed/MEDLINE and adapted for each database. No limits were applied to the publication date, geographic location, recruitment status, or follow-up duration. An English-language filter was applied only to Scopus, and language eligibility was assessed during screening. To assess the potential effect of this restriction, a post hoc language audit was conducted during revision. The Scopus search strategy was rerun without the English-language filter, and the resulting non-English records were assessed against all remaining eligibility criteria. Backward and forward citation searches of the included reports supplemented electronic searches. The search strategy was developed and conducted by the review team. Search comprehensiveness was checked by combining controlled vocabulary and free-text synonyms for bruxism, botulinum toxin, and relevant masticatory muscles; reviewing terminology used in previous reviews and eligible studies; adapting the strategy across databases; and supplementing database searches with trial registry and backward and forward citation searches. An information specialist or librarian was not involved, and the strategy was not externally peer-reviewed. The full search strategy for each source, including all search strings and number of records retrieved, is presented in Supplementary Table S2.

### 2.4. Study Selection

All records were imported into EndNote 20 (Clarivate Analytics, Philadelphia, PA, USA) and duplicates were removed by automated matching, followed by manual verification. Two reviewers (WT and DS) independently screened titles and abstracts based on the eligibility criteria. Records considered potentially eligible by either reviewer proceeded to a full-text assessment, which the same two reviewers performed independently. Disagreements at any stage were resolved by discussion and unresolved disagreements were adjudicated by a third reviewer (AD). Inter-reviewer agreement was not formally quantified because reviewer-level screening decisions were not retained separately after consensus resolution. The reasons for exclusion of the full text were recorded. Trial registrations, protocols, conference abstracts, and journal publications describing the same trial were linked and treated as multiple reports of a single underlying study (Supplementary Table S4). When a potentially eligible report could not be retrieved, retrieval was attempted through institutional library services and author contacts, and the unretrieved reports were documented separately. The post hoc unrestricted Scopus search identified 11 non-English records; none met the remaining eligibility criteria (n = 0 potentially eligible non-English studies).

### 2.5. Data Charting

Data were charted independently by two reviewers (WT and DS) using a form developed by the review team in Microsoft Excel (Microsoft 365; Microsoft Corporation, Redmond, WA, USA). The form was not formally pilot-tested before data charting, which may have increased the risk of inconsistent interpretation of charting fields during the initial extraction stage. The form was therefore refined iteratively during the initial charting process. Refinements clarified the recording of injection strategies, toxin dose, outcome measures, and the distinction between directly reported data, reviewer-calculated values, and review-level interpretations. The final data-charting form is provided in Supplementary Table S5. Charted fields comprised study design, country and setting, sample size, participant characteristics, bruxism type and diagnostic criteria, botulinum toxin preparation, injected muscles, dose per muscle and total dose, number of injection points, injection guidance method, comparator strategy, follow-up duration, outcomes and measurement instruments, reported between-group effect estimates and 95% confidence intervals where available, and adverse events. Directly reported data, values calculated by the review team, and review-level interpretations are recorded in separate fields and distinguished throughout the tables. Registry- and protocol-only records were charted separately from studies reporting the outcome data. When internally inconsistent data were identified within a source report, the report and any linked records were rechecked. If the inconsistency could not be resolved from the available source documents, the conflicting values were reported as such, the affected variable was treated as unclear, and no value was inferred for the synthesis. The original authors were not contacted specifically to resolve these reporting inconsistencies. Discrepancies were resolved through discussion or by consultation with a third reviewer (AD).

### 2.6. Risk-of-Bias Assessment

Although critical appraisal is optional in scoping reviews, the risk of bias was assessed to contextualize the comparative evidence, because the review question concerns the relative effects of alternative injection strategies. The appraisal was restricted to studies reporting outcome data, and registry- and protocol-only records were not appraised. Because RoB 2 is result-specific, the appraisal was intended to contextualize the primary comparative question of this review rather than provide a global study-level judgment across all reported outcomes. Accordingly, one effectiveness result per study was selected using a consistent rule: when a study designated a primary outcome directly relevant to the comparison of expanded versus masseter-only targeting, that result was selected; otherwise, the between-group effectiveness result most directly addressing the review question was used. Other effectiveness outcomes were not formally appraised, and the resulting risk-of-bias judgments should therefore not be generalized to all outcomes reported by each study. RoB 2 was not applied to safety outcomes because it is result-specific and the included studies did not report a sufficiently consistent safety outcome for comparable appraisal. Adverse-event ascertainment and reporting varied, most safety data were descriptive, and one study did not report adverse events. Safety was therefore assessed descriptively using a structured approach that considered whether adverse events were reported, whether reporting was treatment-group specific, event type and resolution, withdrawals related to adverse events, and the ascertainment approach when described. No formal risk-of-bias judgment was assigned to safety outcomes. Although prospective non-randomized comparative interventional studies were eligible, none met the inclusion criteria. Therefore, all appraised studies were randomized trials, and the risk of bias was assessed using the Cochrane RoB 2 tool [23] for the effect of assignment to intervention, using the standard version for parallel-group trials and the crossover variant, including the assessment of period and carryover effects, for the crossover trial. Two reviewers (WT and DS) assessed each study independently and discrepancies were resolved by discussion or, when consensus could not be reached, by a third reviewer (AD). The appraisal did not determine eligibility and no studies were excluded.

### 2.7. Data Synthesis and Presentation

The findings were synthesized descriptively. Meta-analysis was considered inappropriate because studies differed in anatomical injection contrast, bruxism phenotype and assessment method, toxin formulation and dose structure, outcome measures, study design, and follow-up, while several anatomical comparisons were represented by single studies. Studies were classified according to four prespecified injection-strategy categories using the operational rules presented in Supplementary Table S7. Comparisons in which additional muscle targeting also increased the total toxin dose were considered dose-unmatched; therefore, anatomical targeting effects could not be separated from dose escalation. Absolute toxin doses were not compared across formulations because potency units are product-specific and should not be assumed to be interchangeable [24]. Between-group comparisons were prioritized over within-group changes, with study-designated primary outcomes emphasized while retaining other clinically, physiologically, and safety-relevant outcomes. All relevant follow-up time points were charted, and outcomes assessed using different instruments were interpreted separately. Reported between-group effect estimates and 95% confidence intervals were prioritized where available; otherwise, the original summary data and *p*-values were retained without deriving additional measures of precision. Findings were interpreted in relation to the bruxism phenotype and assessment approach without assuming equivalence across phenotypes or assessment methods. Pediatric and adult evidence was to be considered separately if both were identified. Detailed numerical outcomes are presented in Supplementary Table S3.

## 3. Results

**Figure 1:**
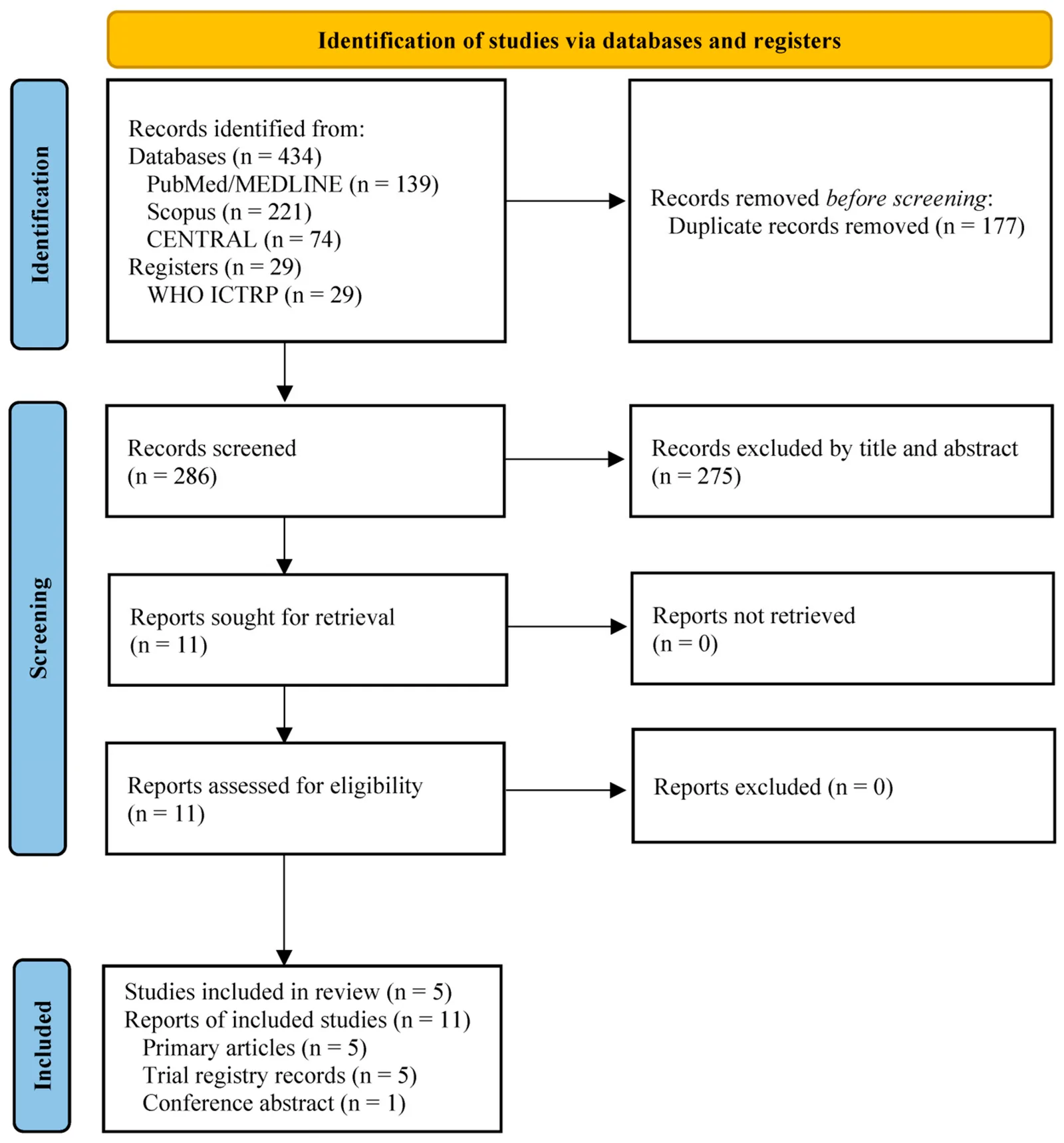
PRISMA 2020 flow diagram of the identification, screening, and inclusion of studies comparing botulinum toxin muscle-targeting strategies for bruxism. CENTRAL, Cochrane Central Register of Controlled Trials; PRISMA, Preferred Reporting Items for Systematic Reviews and Meta-Analyses; WHO ICTRP, World Health Organization International Clinical Trials Registry Platform.

The study selection process is illustrated in Figure 1. A total of 463 records were identified, comprising 434 from the electronic databases (PubMed/MEDLINE, n = 139; Scopus, n = 221; CENTRAL, n = 74) and 29 from the WHO International Clinical Trials Registry Platform. After the removal of 177 duplicates, 286 records were screened by title and abstract, of which 275 were excluded, principally because no masseter-only comparison arm was present, the comparator was a placebo or a non-injection treatment rather than an alternative injection pattern, or the record did not report the original clinical data. Eleven eligible reports were retrieved and assessed, corresponding to five unique studies [11,13,14,15,17]. These 11 reports comprised five primary journal articles, five linked trial registry records, and one conference abstract; companion reports were linked to their parent study and were not counted as additional studies (Supplementary Table S4). Backward and forward citation searches did not identify any additional unique eligible records.

The included studies were published between 2014 and 2025 and conducted in South Korea (2 studies), Australia, Brazil, and Syria. Four were parallel-group randomized trials and one was a placebo-controlled randomized crossover trial [13]. No eligible prospective non-randomized comparative study was identified. Four parallel-group trials compared masseter-only injections with an expanded strategy that included the temporalis, anterior belly of the digastric, or lateral pterygoid, whereas a crossover trial compared three active strategies involving the masseter alone, masseter plus temporalis, and masseter plus temporalis and medial pterygoid [13]. Across all studies, the masseter dose was held constant when additional muscles were added, resulting in higher total toxin doses with broader targeting; therefore, anatomical targeting and total dose did not vary independently. The follow-up period ranged from 4 weeks to 6 months. All five studies were registered, although registration followed the start of enrollment in 3. Across the five studies, 133 participants were randomized, but only 116 (87%) contributed to the comparative analyses; 13 of 35 participants in Cruse et al. [13] and 4 of 24 in Shim et al. [17] were excluded from the relevant analyses after randomization. These exclusions reduced the effective sample size and may have introduced attrition-related bias if exclusion or missingness was associated with treatment assignment or outcomes. This concern was greatest for Cruse et al., which was judged to be at high risk of bias for missing outcome data. Accordingly, the findings from these small trials should be interpreted cautiously. The mean age ranged from 25.8 to 42.1 years, and women constituted 64–83% of the participants in every study. No pediatric study was identified; therefore, no cross-age comparison was required in the synthesis. The bruxism phenotype and assessment approach varied across studies. Three studies specifically enrolled participants with sleep bruxism: Cruse et al. [13] and Shim et al. [17] incorporated instrumental assessment using electromyography or video-polysomnography, whereas Kim et al. [15] used self-reported sleep clenching together with morning jaw stiffness and clinical tooth wear. Da Silva Ramalho et al. [14] included participants with sleep or awake bruxism using clinical and self-reported criteria, while Alzaeem et al. [11] did not distinguish sleep from awake bruxism but required questionnaire, clinical, and electromyographic findings. Thus, three studies incorporated instrumental assessment, whereas two relied primarily on non-instrumental clinical or self-reported criteria. Because these diagnostic groups contained few studies and differed further in outcomes and injection protocols, no formal comparison according to bruxism phenotype or assessment approach was undertaken. The relationship between bruxism phenotype, assessment approach, and comparative findings is summarized in Supplementary Table S6. Coexisting orofacial pain or headache was present in the majority of participants in 3 studies and was an exclusion criterion in another study. Baseline characteristics were comparable between the groups, except for imbalances in body mass index [17] and left lateral pterygoid electromyographic activity (*p* = 0.037) [11]. Overall, the included studies represented a clinically heterogeneous evidence base, with substantial differences in bruxism phenotype and assessment method, coexisting pain or temporomandibular characteristics, injection protocols, outcomes, and follow-up duration. Accordingly, findings were interpreted within each study and injection comparison rather than as directly comparable estimates across trials. The characteristics of the study participants are presented in Table 1.

The injection protocols differed in nearly every technical aspect, including toxin preparation (5 different products), reconstitution concentration (4–10 U per 0.1 mL), needle gauge (22–30), number of masseter injection sites (one–three), and guidance technique, which ranged from anatomical landmarks alone to ultrasonographic or electromyographic targeting of deeper muscles. The total bilateral doses ranged from 30 to 60 U in the masseter-only arms and from 50 to 120 U in the expanded arms, with the increment attributable to the additional muscle ranging from 20 to 60 U. The protocols are detailed in Table 2. These technical differences substantially limit direct comparison across studies because toxin formulation, dose, injection-site distribution, and guidance technique may influence treatment effects independently of muscle selection. Accordingly, effect magnitudes were interpreted primarily within each study rather than as directly comparable across trials. No eligible studies evaluated an alternative non-masseter-only muscle-targeting strategy or another comparative injection approach outside of the identified direct add-on and dose-escalating multimuscle comparisons.

Because every expanded-muscle strategy also increased the total toxin dose, all comparative effectiveness findings were interpreted as dose-unmatched anatomical comparisons. Accordingly, any between-group difference could reflect the additional muscle targeted, the higher total toxin exposure, or both, and was not specifically attributed to anatomical targeting. None of the included studies reported 95% confidence intervals for the review-relevant between-group comparisons. Accordingly, the synthesis retained the reported effect estimates and group summary data where available, together with the original *p*-values, rather than deriving confidence intervals from incomplete published data. No two studies provided sufficiently comparable primary outcomes, measurement methods, and follow-up time points for quantitative pooling. The included studies used heterogeneous primary outcomes, which were predominantly physiological or objective measures; a patient-reported clinical outcome, orofacial pain, was designated as the primary outcome in only one study [14]. Across the reported patient-reported and functional outcomes, no statistically significant between-group difference favoring expanded muscle targeting was detected. In Kim et al. [15], no significant group-by-time interaction was detected for bite force (*p* = 0.852) or masseter thickness (*p* = 0.349), and anterior digastric thickness did not significantly decrease after injection. In da Silva Ramalho et al. [14], no statistically significant between-group differences were detected in pain, satisfaction, or bite force at any assessment through 180 days. Two studies showed an effect confined to the additionally injected muscle: temporalis electromyographic amplitude fell further when the temporalis was injected (*p* = 0.010 during maximal voluntary clenching and *p* = 0.025 during rhythmic masticatory muscle activity), without a change in rhythmic masticatory muscle activity or subjective symptoms [17]. Lateral pterygoid activity fell further when the muscle was injected (*p* < 0.001), without differences in questionnaire scores at any of the four post-treatment assessments through 6 months [11]. In the single dose-escalating trial, the three-muscle protocol produced the largest reduction in Bruxism Index relative to placebo (mean −2.15; *p* = 0.009), but the comparison among the three injection paradigms was a secondary analysis and was not statistically significant (*p* = 0.15), and no subjective outcome differed from placebo in any arm [13]. Safety reporting varied across studies and was absent in one study [11]. In the two parallel-group studies reporting adverse events by treatment group, events occurred only in the expanded-injection groups: two of 10 participants in da Silva Ramalho et al. [14] and two of 12 in Kim et al. [15], with none reported in the corresponding masseter-only groups. Events in the crossover trial were distributed across the arms, and one participant withdrew after injection discomfort [13]. One study reported no significant adverse events at 1 month, although 14 of 20 participants (70%) reported discomfort from reduced masticatory force [17], and one study did not report adverse event data [11]. Given the small samples, generally short follow-up, and inconsistent adverse-event ascertainment, these data are insufficient to establish the comparative safety profile of broader muscle targeting. The findings are summarized in Table 3, and the numerical details are provided in Supplementary Table S3.

Because RoB 2 is specific to an individual result, one effectiveness result per study was appraised according to the selection criteria described in Section 2.6, and the result assessed for each study is identified in Table 4. Four of the five results were judged to raise some concerns, and one was judged to be at a high risk of bias [13], reflecting the complete-case analysis of 22 of the 35 randomized participants (63%) for a comparison that was itself secondary. The domain-level judgments were uneven. Deviations from the intended interventions posed a low risk in all five results. All five results raised some concerns regarding the selection of the reported results because no pre-specified statistical analysis plan was accessible, and registration followed the start of enrollment in three studies. The randomization process raised some concerns in four studies in which sequence generation or allocation concealment was incompletely reported and was judged as low risk only when web-based randomization with opaque sealed envelopes was described [14]. Missing outcome data raised concerns in two studies, seriously in one [13] and to a lesser degree in another, where 4 of 24 randomized participants (17%) were excluded from the analysis [17]. Outcome measurement raised some concerns where effort-dependent measures were obtained without reported assessor blinding [15] but was judged at low risk where outcome assessment was adequately blinded or the measurement process was unlikely to be influenced by knowledge of the intervention assignment. The domain-level judgments are presented in Table 4. Overall, these result-specific risk-of-bias concerns reduce confidence in the comparative findings and support interpreting them as preliminary.

## 4. Discussion

### 4.1. Principal Findings and Interpretation of Comparative Injection Strategies

**Figure 2:**
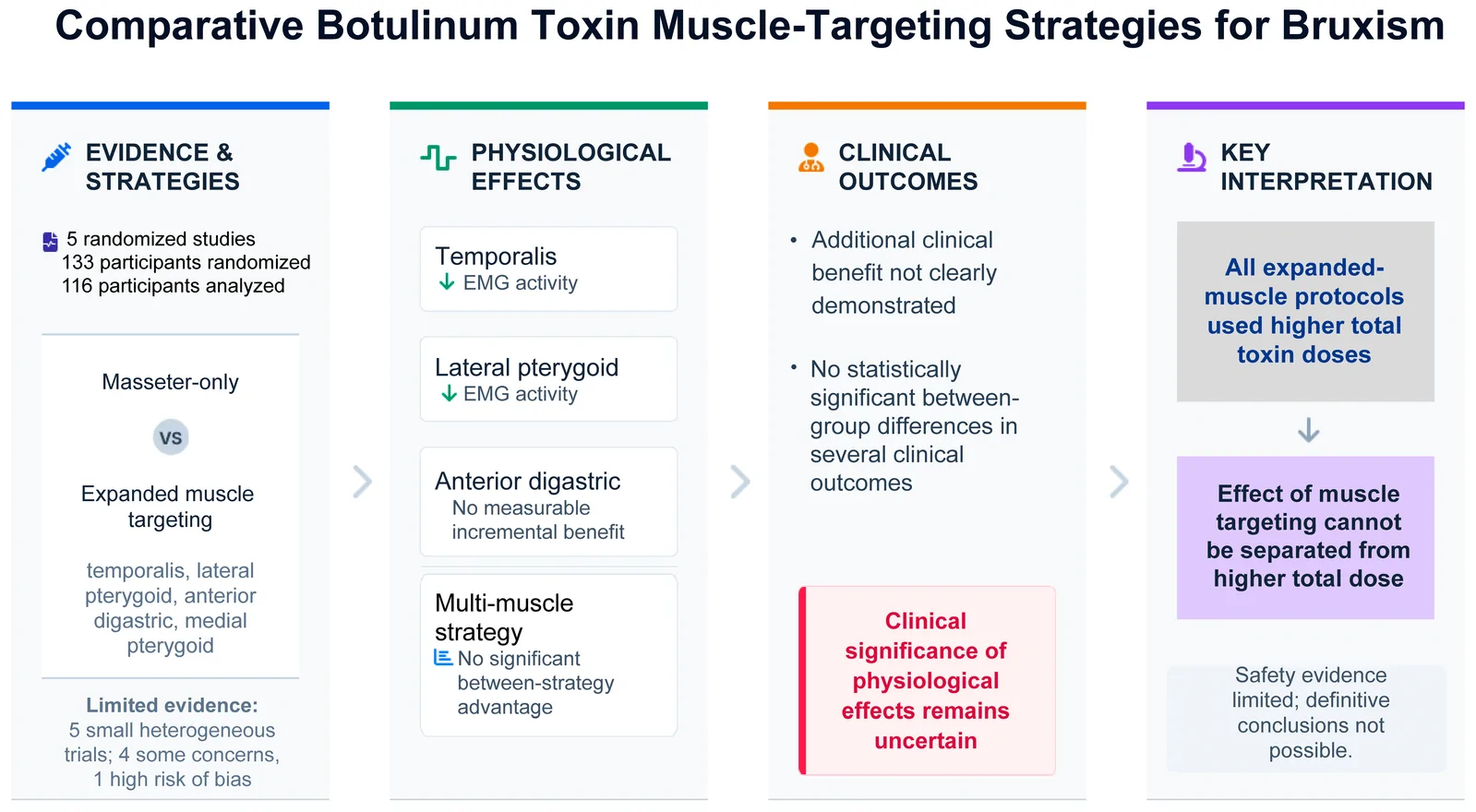
Comparative evidence on botulinum toxin muscle-targeting strategies for bruxism. Five randomized comparative studies evaluated masseter-only injection versus expanded muscle targeting involving the temporalis, lateral pterygoid, anterior digastric, or medial pterygoid. Additional clinical benefit from broader targeting was not clearly demonstrated, although muscle-specific reductions in electromyographic activity were observed for the temporalis and lateral pterygoid in individual studies. All expanded-muscle protocols used higher total toxin doses, preventing separation of anatomical targeting effects from dose escalation. The evidence base was limited by small samples, heterogeneous outcomes and protocols, short follow-up, and incomplete safety reporting; therefore, definitive conclusions regarding comparative effectiveness or safety cannot be drawn. EMG, electromyography.

The available comparative evidence was limited to five small, clinically and methodologically heterogeneous trials, and all expanded strategies increased the total toxin dose while the masseter dose was held constant. Interpretation is further limited by post-randomization exclusions in two trials, because 17 of 133 randomized participants did not contribute to the relevant comparative analyses, potentially reducing precision and introducing attrition-related bias. Consequently, the effect of anatomical expansion could not be separated from that of dose escalation, and any between-group difference could not be attributed specifically to the additional muscle(s). Given the small, heterogeneous evidence base, dose confounding, and risk-of-bias concerns, the comparative findings should be regarded as preliminary. Within these limitations, additional clinical benefit from broader targeting was not clearly demonstrated, although some studies reported muscle-specific physiological effects. The principal comparative findings and their clinical interpretations are summarized in Figure 2.

Across the anatomical comparisons, broader targeting produced muscle-specific physiological changes in some studies, particularly reduced temporalis and lateral pterygoid electromyographic activity [11,17]. In these studies, however, the muscle-specific changes were not accompanied by statistically significant between-group differences in the corresponding patient-reported outcomes [11,17]. In the other direct add-on trials, no statistically significant between-group differences were detected in pain, treatment satisfaction, or bite force [14], or in bite force or masseter thickness [15]. In the dose-escalating multi-muscle trial, the most extensive strategy showed the largest numerical reduction in bruxism activity, but the between-strategy comparison was not statistically significant [13]. These findings should not be interpreted as evidence of equivalence or absence of benefit because the studies were small, heterogeneous, and dose-unmatched. Rather, the incremental clinical value of broader muscle targeting remains uncertain. Assessment of the magnitude, precision, and clinical importance of these differences was limited because none of the included studies reported 95% confidence intervals for the review-relevant between-group comparisons, and validated bruxism-specific thresholds for clinically meaningful change were unavailable. One possible explanation for the apparent dissociation between physiological and clinical outcomes, derived primarily from sleep-bruxism evidence, is that botulinum toxin may reduce contraction intensity in injected muscles without substantially altering the underlying bruxism motor events. In Shim et al. [17], botulinum toxin reduced peak electromyographic amplitude in the injected muscles without reducing the frequency, number of bursts, or duration of rhythmic masticatory muscle activity. This mechanistic interpretation is hypothesis-generating, should be considered in the context of sleep bruxism, and should not be extrapolated to awake or unspecified bruxism because the available comparative trials were not designed to test this mechanism directly. Whether attenuation of additional muscles provides a clinically meaningful benefit therefore remains unresolved.

Previous systematic reviews have addressed the overall efficacy and safety of botulinum toxin for bruxism, rather than the comparative effectiveness of different muscle-targeting strategies. Ågren et al. [10] evaluated the effects on bite force and electromyographic activity and included studies using both masseter-only and masseter-plus-temporalis injections but did not compare these strategies directly. Similarly, Sendra et al. [16] reviewed the clinical outcomes across several injection protocols and noted that most studies evaluated a single injection method, with only one study directly comparing masseter-only with masseter-plus-temporalis treatment. Chen et al. [12] subsequently examined the overall efficacy, treatment duration, and dose–response relationships, but did not synthesize outcomes according to the muscle-targeting strategy. Therefore, this review addresses a distinct evidence gap by directly mapping whether extending injections beyond the masseter provides incremental benefits over the masseter-only treatment. Although previous reviews suggest that botulinum toxin can reduce muscle activity, bite force, pain, or other bruxism-related outcomes, these overall treatment effects do not establish whether injecting additional muscles provides greater clinical benefits.

### 4.2. Anatomical Rationale for Additional Muscle Targeting

Bruxism involves several masticatory muscles, providing an anatomical rationale for considering targets beyond the masseter [7,8,9]. The included comparative studies evaluated additional targeting of the temporalis, lateral pterygoid, anterior digastric, or medial pterygoid [11,13,14,15,17]. However, each additional target was examined in only limited comparative evidence, with substantial heterogeneity in bruxism assessment, outcomes, and injection protocols. Moreover, every expanded strategy used a higher total toxin dose, preventing the effects of muscle selection from being separated from dose escalation. Therefore, anatomical plausibility should not be interpreted as evidence of incremental clinical effectiveness, and the available evidence is insufficient to determine whether any specific muscle should routinely be added to masseter injection.

### 4.3. Safety and Adverse Effects of Masticatory-Muscle Injection

The safety evidence from the included comparative studies was limited by small sample sizes, generally short follow-up, and inconsistent adverse-event ascertainment. Reported events were predominantly transient, but adverse-event data were not reported in Alzaeem et al. [11], and the available evidence is insufficient to establish the comparative safety profile of expanded muscle targeting. Because no formal risk-of-bias assessment was applied to safety outcomes, these findings should be regarded as descriptive and may be affected by incomplete or selective adverse-event ascertainment and reporting. Adverse events were systematically assessed in Cruse et al. [13], whereas Shim et al. [17] reported no significant adverse events despite 14 of 20 participants reporting discomfort from reduced masticatory force and 3 reporting masticatory difficulty. In the two parallel-group studies that reported adverse events separately by treatment group, events occurred only in the expanded-injection groups. Because these groups also received higher total toxin doses, the effects of muscle selection and dose could not be distinguished.

In contrast, indirect safety evidence derived mainly from cosmetic masseter hypertrophy populations has described transient chewing weakness, injection-site pain or bruising, altered facial expression or contour, xerostomia, and paradoxical masseter bulging after botulinum toxin injection [25,26,27,28]. A separate placebo-controlled bruxism trial also reported smile asymmetry [29]. Because these studies differed in indication, population, treatment protocol, and dosing regimen, their findings were considered contextual only and were not used to estimate the comparative safety of the injection strategies evaluated in this review.

Long-term effects of repeated botulinum toxin exposure were not evaluated in the included comparative studies. Evidence from other populations has reported persistent reductions in masseter muscle volume after repeated injections and possible mandibular bone changes, although findings for bone outcomes have been inconsistent [30,31,32,33,34,35]. These studies were largely observational and involved populations, indications, and treatment regimens that differed from those included in this review. Accordingly, they provide contextual evidence only and do not establish a causal long-term risk or the comparative safety of broader muscle targeting in patients with bruxism.

### 4.4. Limitations, Evidence Gaps, and Future Directions

The principal limitations of this review were the small evidence base and sample sizes, systematic dose imbalance between expanded and masseter-only strategies, restriction to English-language reports, and absence of Embase. First, only five small comparative studies met the eligibility criteria, and all expanded strategies used higher total toxin doses than their masseter-only comparators, preventing separation of anatomical targeting from dose escalation. Substantial heterogeneity in bruxism assessment, targeted muscles, toxin formulations, injection protocols, outcome measures, and follow-up further precluded meta-analysis. Accordingly, this review maps the available evidence rather than establishing comparative effectiveness. Second, eligibility was restricted to English-language reports because reliable translation resources were unavailable. Although a post hoc language audit of the unrestricted Scopus search identified no non-English study that otherwise met the eligibility criteria, the English-language restriction remains a potential source of language bias and may affect the completeness and generalizability of the evidence map. Although four information sources were systematically searched, and citation searching was undertaken, the search strategy was not externally peer-reviewed by an information specialist or librarian. Embase was not searched because institutional access was unavailable, which may have resulted in the omission of relevant pharmacologic or biomedical intervention studies. Third, bruxism was defined and assessed inconsistently across the included studies, and consensus-based assessment frameworks distinguishing sleep from awake bruxism and different levels of assessment certainty were applied variably or not at all [22]. Self-reporting has limited diagnostic accuracy for sleep bruxism compared to instrumental assessment [36,37], whereas electromyographic assessment itself remains heterogeneous, with numerous thresholds used to define bruxism-related events [38]. Sleep bruxism also exhibits substantial night-to-night variability, which complicates the interpretation of treatment-related changes based on limited recordings [39]. Fourth, the heterogeneity in toxin formulation, dose, injection sites, and guidance methods further limited cross-study interpretation; moreover, botulinum toxin type A formulations are not interchangeable, and their potency units are product-specific [24]. Fifth, blinding may be difficult to maintain, because reductions in muscle activity, bite force, or visible muscle bulk may reveal treatment allocation, particularly for subjective outcomes [40]. In addition, the data-charting form was not formally pilot-tested, which may have introduced inconsistency in the interpretation of fields during initial extraction, although independent duplicate charting and consensus procedures were used to mitigate this risk. RoB 2 was applied to one selected effectiveness result per study; therefore, risk of bias in other reported outcomes may differ and was not formally characterized. Finally, the included studies had small samples, generally short follow-up periods, heterogeneous primary outcomes, and incomplete reporting of some methodological and safety domains, limiting the precision and generalizability of the comparative evidence.

These limitations highlight several avenues for future studies. First, future comparative trials should distinguish the effect of anatomical targeting from that of total toxin dose by using dose-controlled strategies where feasible. The same toxin formulation should be used across groups because potency units are product-specific and should not be assumed equivalent between products [24]. Second, participants should be characterized using standardized definitions that distinguish sleep bruxism from awake bruxism, and these phenotypes should preferably be analyzed separately. For sleep bruxism, instrumental assessment should use sufficiently robust recording protocols, and where feasible, repeated-night measurements should account for night-to-night variability [38,39]. Third, dose-controlled trials should assess objective physiological and patient-important outcomes together and prespecify a patient-important primary outcome, such as orofacial pain intensity in symptomatic participants. Between-group effect estimates should be reported with 95% confidence intervals, and a clinically meaningful between-group difference for the selected primary outcome should be defined a priori and justified according to the outcome measure and target population. Where possible, this threshold should be based on bruxism-specific evidence; however, validated thresholds for clinically meaningful change in bruxism are currently lacking, and thresholds derived from temporomandibular disorder populations should not be assumed to apply directly [41]. Appropriate statistical methods and handling of missing data should also be prespecified [42]. Fourth, the success of the blinding should be assessed and reported where feasible [40]. Longer follow-up and studies with repeated treatment cycles are also needed to evaluate durability, chewing function, bite force, muscle thickness, facial changes, and potential mandibular bone effects. Fifth, direct comparisons of anatomical landmarks, electromyography, and ultrasound-guided injections may further clarify whether targeting accuracy influences effectiveness or safety [43]. Finally, future studies should consider the cost-effectiveness, patient preferences, and clinical outcomes.

## 5. Conclusions

The available comparative findings are preliminary and insufficient to determine whether broader botulinum toxin injection strategies provide greater or lesser clinical benefit than masseter-only treatment for bruxism. The evidence base is small and heterogeneous, with risk-of-bias concerns, and all expanded strategies used higher total toxin doses; therefore, the observed effects cannot isolate the contribution of additional muscle targeting from dose escalation. No pediatric evidence was identified, and long-term safety remains uncertain because repeated-treatment effects were not evaluated in the included comparative studies. Accordingly, the comparative value of broader muscle targeting remains uncertain.

## Figures and Tables

**Table 1:** Characteristics of the included studies and participants.

Study; Country; Design and Setting	Bruxism Population and Diagnostic Criteria	Participants Analyzed	Relevant Baseline Profile	Follow-Up and Registration
Alzaeem et al., 2025 [11] Syria; double-blind, randomized, parallel-group, active-controlled trial; Damascus University, Jan–Nov 2024	Bruxism questionnaire score >= 50%; increased masseter and lateral pterygoid EMG activity; clinical dental erosion. Eligibility specified age 18–40 years (abstract states 18–45 years).	41 assessed; 30 randomized and analyzed (15/group). Mean age 26.94 +/− 8.40 years; 83.33% female.	Groups were demographically similar. Baseline left lateral pterygoid EMG differed between groups (*p* = 0.037); baseline questionnaire scores did not differ significantly.	EMG: baseline and 2 weeks. Questionnaire: baseline, 2 weeks, 6 weeks, 3 months, and 6 months. ISRCTN13377883; registered 20 Dec 2024 after the reported Jan–Nov 2024 study period.
Kim et al., 2024 [15] South Korea; randomized parallel clinical trial; Yonsei University Dental Hospital	Sleep bruxism based on clenching during sleep >= 3 times/week, morning jaw stiffness, and clinical tooth wear; prior splint or BoNT treatment and TMD were excluded.	24 randomized and analyzed (12/group). Mean age 34.3 +/− 6.8 years; 18 female/6 male.	No significant between-group differences in age, sex, baseline bite force, or baseline masseter thickness.	Baseline, 4 weeks, and 8 weeks. KCT0008721. Study period NR; IRB approval was granted on 23 Feb 2022. Registration timing was not stated in the article.
da Silva Ramalho et al., 2023 [14] Brazil; two-arm, 1:1, patient-blinded parallel RCT; university dental clinic, Feb–Nov 2019	Adults with sleep or awake bruxism for >=6 months plus bilateral myofascial pain at rest, clenching, or palpation; TMD and current splint use were excluded.	24 assessed; 20 randomized and analyzed (10/group). Mean age: 39 years (masseter) and 37 years (masseter + temporalis); each group 8 female/2 male.	No statistically significant baseline differences in pain or posterior bite force were reported.	Baseline and 15, 90, 120, and 180 days. ReBEC: RBR-39ght5; WHO Universal Trial Number: U1111-1235-5386.
Cruse et al., 2022 [13] Australia; double-blind, placebo-controlled randomized crossover trial; Royal Melbourne Hospital and referrals, 2017–2020	Sleep bruxism according to ICSD-R criteria and baseline Bruxism Index (BI) > 5 based on three nights of home surface EMG.	41 assessed; 35 randomized; 22 complete cases analyzed: masseter n = 6, masseter + temporalis n = 7, three-muscle n = 9. Mean age 42.1 ± 13.98 years. Sex distribution was internally inconsistent: the article text reported 14 female/8 male, whereas the subgroup counts summed to 11 female/11 male.	Mean baseline BI 8.29 +/− 2.88. Headache disorders in 18/22; previous occlusal splint use in 18/22.	Active and placebo injections separated by 20 weeks; outcomes at 4 and 12 weeks after each phase. ACTRN12618001430224; registered 27 Aug 2018 after registry-listed enrollment began.
Shim et al., 2014 [17] South Korea; randomized parallel comparative trial with blinded outcome scoring; Yonsei University Dental Hospital	Clinical sleep bruxism: tooth grinding >= 3 nights/week, morning jaw stiffness, and tooth wear despite oral splint treatment; video-polysomnography used to confirm/analyze RMMA.	24 randomized; 20 analyzed (10/group). Mean age 25.8 +/− 5.1 years; 13 female/7 male.	Masticatory muscle pain in 15/20; TMJ click in 11/20; all had morning jaw stiffness. BMI differed at baseline (*p* = 0.019).	Baseline and 4 weeks. NCT01336439; registered 4 Apr 2011 after enrollment began in March 2011.

Note. BI, Bruxism Index; BoNT, botulinum toxin; EMG, electromyography; ICSD-R, International Classification of Sleep Disorders-Revised; RMMA, rhythmic masticatory muscle activity; RCT, randomized controlled trial; TMD, temporomandibular disorder; TMJ, temporomandibular joint; BMI, body mass index. Across the five studies, 133 participants were randomized and 116 were included in the final analyses. The reported numbers assessed for eligibility summed to 154, although recruitment terminology varied across studies. In Cruse et al., the overall text reports 14 female and 8 male participants, whereas the subgroup counts in Table 1 sum to 11 females and 11 males. No sex-distribution value was adjudicated; the discrepancy was retained as unresolved and was not used in the comparative synthesis.

**Table 2:** Botulinum toxin formulations and comparative muscle-targeting strategies.

Study	BoNT-A Preparation and Guidance	Masseter-Only Strategy	Expanded-Muscle Strategy	Dose/Technique Interpretation
Alzaeem et al., 2025 [11]	TOXTA (JETEMA); reconstituted with 2 mL saline (50 U/mL; 5 U/0.1 mL). Masseter located anatomically; lateral pterygoid targeted with an injectable EMG needle.	15 U/side into masseter (5 U at each of 3 sites); total bilateral dose 30 U. Entry depth 4–6 mm.	Same masseter dose plus lateral pterygoid 10 U/side; total 50 U. Deep extraoral access, 25–30 mm, approximately 30 degrees to the occlusal plane.	Expanded arm received 20 U more. Total dose was not matched; the lateral pterygoid-specific effect cannot be separated from dose escalation.
Kim et al., 2024 [15]	Nabota (Daewoong); 100 U diluted in 2.5 mL saline (4 U/0.1 mL); 27-gauge, 0.5-inch needle; ultrasound guidance with Doppler for the digastric region.	25 U/side into masseter; total 50 U. Ultrasound-guided dual-plane injection relative to the deep inferior tendon; number of puncture sites NR.	Same masseter dose plus anterior belly of digastric 10 U/side; total 70 U. Targeted in the middle third under ultrasound/Doppler guidance. Authors proposed a 7.3–8.3 mm target depth.	Expanded arm received 20 U more. Total dose was not matched.
da Silva Ramalho et al., 2023 [14]	Botulift (Medytox); 100 U vial reconstituted with 1 mL saline (10 U/0.1 mL); anatomical landmark injection with a 22-gauge needle. The article reports 1 mL injected at each point, but this conflicts with the stated concentration and dose; injection volume per point is therefore unclear (see note).	30 U/side into masseter (10 U at each of 3 sites); total 60 U.	Same masseter dose plus temporalis 20 U/side (10 U at each of 2 sites); total 100 U.	Expanded arm received 40 U more. Total dose was not matched.
Cruse et al., 2022 [13]	Botox/onabotulinumtoxinA (Allergan); 100 U in 2 mL saline (5 U/0.1 mL). Masseter and medial pterygoid injections were EMG-guided with a 26-gauge needle; temporalis was palpation-guided with a 30-gauge needle.	30 U/side into masseter at one site; total 60 U.	Two-muscle arm: same masseter dose + temporalis 15 U/side (5 U at each of 3 sites); total 90 U. Three-muscle arm: same + medial pterygoid 15 U/side at one site; total 120 U. Medial pterygoid approached extraorally/submandibularly to 10–15 mm.	Number of muscles and total dose escalated together (60, 90, and 120 U); anatomical and dose effects are inseparable.
Shim et al., 2014 [17]	Neuronox (Medytox); 200 U in 4 mL saline (5 U/0.1 mL); 29-gauge, 0.5-inch needle; sites identified by palpation during clenching.	25 U/side into masseter across 3 sites; total 50 U.	25 U/side into masseter plus 25 U/side into temporalis, each across 3 sites; total 100 U.	Expanded arm received 50 U more. Total dose was not matched.

Note. BoNT-A unit potency is formulation-specific; doses should be interpreted within each study rather than directly converted across products. Across all five studies, expanded muscle targeting was accompanied by a higher total toxin dose. In da Silva Ramalho et al., the article states that 1 mL was injected at each facial point; this is internally inconsistent with a 100-U vial reconstituted in 1 mL and the reported dose of 10 U per point. The administered volume per point was therefore recorded as unclear and was not inferred or used in the synthesis. The reported dose of 10 U per injection point and the number of injection points were retained as explicitly reported in the article. NR, not reported.

**Table 3:** Comparative effectiveness and safety of botulinum toxin muscle-targeting strategies.

Study	Outcomes and Assessment Times	Between-Group Comparative Findings	Safety Findings
Alzaeem et al., 2025 [11]	Primary: masseter and lateral pterygoid EMG at baseline and 2 weeks. Secondary: 4-item bruxism questionnaire at baseline, 2 weeks, 6 weeks, 3 months, and 6 months.	Questionnaire scores did not differ significantly between groups at 2 weeks (*p* = 1.000), 6 weeks (*p* = 0.372), 3 months (*p* = 0.624), or 6 months (*p* = 0.831). Masseter EMG did not differ between groups at 2 weeks. Lateral pterygoid EMG favored the expanded arm at 2 weeks (both sides *p* < 0.001), although left-side baseline EMG differed (*p* = 0.037).	Adverse events were not reported for the trial.
Kim et al., 2024 [15]	Bite force and masseter/anterior digastric thickness at baseline, 4 weeks, and 8 weeks; subjective reduction in sleep bruxism and adverse effects.	No significant group-by-time interaction for bite force (*p* = 0.852) or masseter thickness (*p* = 0.349). Anterior digastric thickness did not significantly decrease after injection. All participants in both groups reported decreased sleep bruxism.	Masseter-only: no adverse effects reported. Expanded arm: 2/12 (16.7%) reported temporary difficulty closing the mouth; symptoms resolved by 8 weeks.
da Silva Ramalho et al., 2023 [14]	Primary: orofacial pain VAS. Secondary: treatment satisfaction VAS and left posterior bite force. Baseline and 15, 90, 120, and 180 days.	No significant between-group differences in pain, satisfaction, or bite force at any follow-up. Both groups improved from baseline in pain; bite force reduction persisted through 120 days but not 180 days.	Two women in the masseter + temporalis arm developed marked reduction in masticatory efficiency and smile asymmetry, respectively; both lasted several weeks. No event was reported in the masseter-only arm.
Cruse et al., 2022 [13]	Primary: surface-EMG BI comparing active treatment with placebo at 4 and 12 weeks. Secondary: differences among injection groups; bruxism symptoms, pain, headache impact, sleepiness, and adverse events.	Overall active treatment reduced BI versus placebo at 4 weeks (mean difference −1.66; *p* = 0.003), but not at 12 weeks (*p* = 0.58). The three-muscle arm had the largest 4-week reduction versus placebo (−2.15; *p* = 0.009); masseter-only *p* = 0.287 and two-muscle *p* = 0.073. There was no statistically significant difference among injection groups (F(2,21) = 2.11; *p* = 0.15), and subjective outcomes did not differ.	Reported events included transient chewing weakness/fatigue (4 participants: 2 three-muscle, 1 two-muscle, 1 masseter-only), lower facial weakness for 10 days (two-muscle), mild bruising (masseter-only), and withdrawal after injection discomfort (three-muscle).
Shim et al., 2014 [17]	Video-polysomnographic RMMA index and episode characteristics; peak EMG amplitude during maximal voluntary clenching and RMMA; subjective grinding, morning stiffness, and masticatory force at 4 weeks.	No between-group differences in RMMA frequency, bursts, sound, or episode duration. Adding temporalis produced greater temporalis EMG reductions during maximal voluntary clenching (*p* = 0.010) and RMMA (*p* = 0.025), but no masseter EMG or subjective-symptom advantage.	Fourteen of 20 reported discomfort from reduced masticatory force; 3 reported masticatory difficulty. Mean perceived force reduction was 30.0% in masseter-only and 40.5% in expanded treatment (*p* = 0.208). No significant serious adverse effect was reported at 1 month.

Note. Interpretation prioritizes between-group evidence. Improvement from baseline in both groups does not by itself demonstrate benefit from adding another muscle. All expanded-muscle comparisons were dose-unmatched; therefore, between-group differences cannot isolate the effect of anatomical targeting from that of increased total toxin dose. BI, Bruxism Index; EMG, electromyography; RMMA, rhythmic masticatory muscle activity; VAS, visual analog scale.

**Table 4:** Risk-of-bias assessment of the selected review-relevant effectiveness result from each included randomized trial.

Study	Result Assessed	Randomization Process	Deviations from Intended Interventions	Missing Outcome Data	Measurement of the Outcome	Selection of the Reported Result	Period/Carryover Effects	Overall Risk of Bias
Alzaeem et al., 2025 [11]	Lateral pterygoid EMG activity at 2 weeks, between-group comparison	Some concerns	Low risk	Low risk	Low risk	Some concerns	Not applicable	Some concerns
Kim et al., 2024 [15]	Bite force through 8 weeks, group-by-time comparison	Some concerns	Low risk	Low risk	Some concerns	Some concerns	Not applicable	Some concerns
da Silva Ramalho et al., 2023 [14]	Orofacial pain VAS through 180 days, between-group comparison	Low risk	Low risk	Low risk	Low risk	Some concerns	Not applicable	Some concerns
Cruse et al., 2022 [13]	Change in Bruxism Index at 4 weeks, comparison among active injection groups	Some concerns	Low risk	High risk	Low risk	Some concerns	Some concerns	High risk
Shim et al., 2014 [17]	RMMA index at 4 weeks, between-group comparison	Some concerns	Low risk	Some concerns	Low risk	Some concerns	Not applicable	Some concerns

Note. RoB 2 judgments apply to the specific effectiveness result identified in the “Result assessed” column and should not be interpreted as study-level judgments. Risk of bias was assessed for the effect of assignment to intervention. Standard RoB 2 was used for parallel trials, whereas the crossover variant was applied to Cruse et al., including period and carryover effects. Cruse et al. analyzed 22 of 35 randomized participants, resulting in high risk of bias for missing outcome data and an overall high risk of bias. BI, Bruxism Index; EMG, electromyography; RMMA, rhythmic masticatory muscle activity; VAS, visual analog scale.

## Data Availability

All data supporting the findings of this review are available within the manuscript and its supplementary materials.

## Supplementary Materials

The following supporting information can be downloaded at https://www.mdpi.com/article/10.3390/jcm15197712/s1: Table S1: Preferred Reporting Items for Systematic Reviews and Meta-Analyses Extension for Scoping Reviews (PRISMA-ScR) checklist; Table S2: Full search strategy; Table S3: Detailed numerical data for review-relevant efficacy and safety outcomes; Table S4: Linked reports and trial registrations for the included studies; Table S5: Final data-charting form used for the scoping review; Table S6: Bruxism phenotype, assessment approach, and comparative findings of the included studies; Table S7: Prespecified classification rules for comparative botulinum toxin injection strategies.

## Abbreviations

The following abbreviations are used in this manuscript:

CENTRAL	Cochrane Central Register of Controlled Trials
EMG	Electromyography
HIT-6	Headache Impact Test-6
INPLASY	International Platform of Registered Systematic Review and Meta-analysis Protocols
JBI	Joanna Briggs Institute
PCC	Population–Concept–Context
PRISMA-ScR	Preferred Reporting Items for Systematic Reviews and Meta-Analyses Extension for Scoping Reviews
RMMA	Rhythmic masticatory muscle activity
RoB 2	Cochrane Risk of Bias 2 tool
VAS	Visual analog scale
WHO ICTRP	World Health Organization International Clinical Trials Registry Platform
