# Supplementary material for: Comparative Botulinum Toxin Injection Strategies Beyond the Masseter for Bruxism: A Scoping Review
Source: J Clin Med. 2026 Oct 6;15(19):7712. doi: 10.3390/jcm15197712 (PMC13665912; doi:10.3390/jcm15197712)
Supplement: Supplementary file 1 [file jcm-15-07712-s001.zip › jcm-4582020-supplementary.pdf]

Supplementary Table S1. Preferred Reporting Items for Systematic reviews and Meta-Analyses extension for Scoping Reviews (PRISMA-ScR) Checklist

SECTION	ITEM	PRISMA-ScR CHECKLIST ITEM	REPORTED ON PAGE #
**TITLE**			
Title	1	Identify the report as a scoping review.	1
**ABSTRACT**			
Structured summary	2	Provide a structured summary that includes (as applicable): background, objectives, eligibility criteria, sources of evidence, charting methods, results, and conclusions that relate to the review questions and objectives.	2
**INTRODUCTION**			
Rationale	3	Describe the rationale for the review in the context of what is already known. Explain why the review questions/objectives lend themselves to a scoping review approach.	2-3
Objectives	4	Provide an explicit statement of the questions and objectives being addressed with reference to their key elements (e.g., population or participants, concepts, and context) or other relevant key elements used to conceptualize the review questions and/or objectives.	3-4
**METHODS**			
Protocol and registration	5	Indicate whether a review protocol exists; state if and where it can be accessed (e.g., a Web address); and if available, provide registration information, including the registration number.	3
Eligibility criteria	6	Specify characteristics of the sources of evidence used as eligibility criteria (e.g., years considered, language, and publication status), and provide a rationale.	4-5
Information sources*	7	Describe all information sources in the search (e.g., databases with dates of coverage and contact with authors to identify additional sources), as well as the date the most recent search was executed.	5
Search	8	Present the full electronic search strategy for at least 1 database, including any limits used, such that it could be repeated.	5
Selection of sources of evidence†	9	State the process for selecting sources of evidence (i.e., screening and eligibility) included in the scoping review.	5
Data charting process‡	10	Describe the methods of charting data from the included sources of evidence (e.g., calibrated forms or forms that have been tested by the team before their use, and whether data charting was done independently or in duplicate) and any processes for obtaining and confirming data from investigators.	5-6
Data items	11	List and define all variables for which data were sought and any assumptions and simplifications made.	5-6
Critical appraisal of individual sources of evidence§	12	If done, provide a rationale for conducting a critical appraisal of included sources of evidence; describe the methods used and how this information was used in any data synthesis (if appropriate).	6
Synthesis of results	13	Describe the methods of handling and summarizing the data that were charted.	6-7
**RESULTS**			
Selection of sources of evidence	14	Give numbers of sources of evidence screened, assessed for eligibility, and included in the review, with reasons for exclusions at each stage, ideally using a flow diagram.	7-8
Characteristics of sources of evidence	15	For each source of evidence, present characteristics for which data were charted and provide the citations.	8-11
Critical appraisal within sources of evidence	16	If done, present data on critical appraisal of included sources of evidence (see item 12).	15-16

SECTION	ITEM	PRISMA-ScR CHECKLIST ITEM	REPORTED ON PAGE #
Results of individual sources of evidence	17	For each included source of evidence, present the relevant data that were charted that relate to the review questions and objectives.	12-15
Synthesis of results	18	Summarize and/or present the charting results as they relate to the review questions and objectives.	13-16
**DISCUSSION**			
Summary of evidence	19	Summarize the main results (including an overview of concepts, themes, and types of evidence available), link to the review questions and objectives, and consider the relevance to key groups.	17-20
Limitations	20	Discuss the limitations of the scoping review process.	20-21
Conclusions	21	Provide a general interpretation of the results with respect to the review questions and objectives, as well as potential implications and/or next steps.	21
**FUNDING**			
Funding	22	Describe sources of funding for the included sources of evidence, as well as sources of funding for the scoping review. Describe the role of the funders of the scoping review.	22

JBI = Joanna Briggs Institute; PRISMA-ScR = Preferred Reporting Items for Systematic reviews and Meta-Analyses extension for Scoping Reviews.

* Where *sources of evidence* (see second footnote) are compiled from, such as bibliographic databases, social media platforms, and Web sites.

† A more inclusive/heterogeneous term used to account for the different types of evidence or data sources (e.g., quantitative and/or qualitative research, expert opinion, and policy documents) that may be eligible in a scoping review as opposed to only studies. This is not to be confused with *information sources* (see first footnote).

‡ The frameworks by Arksey and O'Malley (6) and Levac and colleagues (7) and the JBI guidance (4, 5) refer to the process of data extraction in a scoping review as data charting.

§ The process of systematically examining research evidence to assess its validity, results, and relevance before using it to inform a decision. This term is used for items 12 and 19 instead of "risk of bias" (which is more applicable to systematic reviews of interventions) to include and acknowledge the various sources of evidence that may be used in a scoping review (e.g., quantitative and/or qualitative research, expert opinion, and policy document).

Supplementary Table S2. The full search strategy

Database	Search strings	Records
PubMed	("Bruxism"[Mesh] OR "Sleep Bruxism"[Mesh] OR brux*[tiab] OR "teeth grinding"[tiab] OR "tooth grinding"[tiab] OR "teeth clenching"[tiab] OR "tooth clenching"[tiab] OR "jaw clenching"[tiab] OR "rhythmic masticatory muscle activity"[tiab] OR "rhythmic masticatory muscle activities"[tiab] OR RMMA[tiab]) AND ("Botulinum Toxins"[Mesh] OR "Botulinum Toxins, Type A"[Mesh] OR "botulinum toxin"[tiab] OR "botulinum toxins"[tiab] OR "botulinum neurotoxin"[tiab] OR "botulinum neurotoxins"[tiab] OR "botulinum toxin type A"[tiab] OR "botulinum neurotoxin type A"[tiab] OR botox[tiab] OR dysport[tiab] OR xeomin[tiab] OR BoNT[tiab] OR BoNTA[tiab] OR "BoNT-A"[tiab] OR BTX[tiab] OR BTXA[tiab] OR "BTX-A"[tiab] OR BTA[tiab] OR onabotulinumtoxinA[tiab] OR abobotulinumtoxinA[tiab] OR incobotulinumtoxinA[tiab] OR prabotulinumtoxinA[tiab] OR letibotulinumtoxinA[tiab] OR daxibotulinumtoxinA[tiab] OR rimabotulinumtoxinB[tiab]) AND ("Masticatory Muscles"[Mesh] OR "Masseter Muscle"[Mesh] OR "Temporal Muscle"[Mesh] OR "Pterygoid Muscles"[Mesh] OR masseter*[tiab] OR temporalis[tiab] OR "temporalis muscle"[tiab] OR "temporalis muscles"[tiab] OR "temporal muscle"[tiab] OR "temporal muscles"[tiab] OR pterygoid*[tiab] OR digastric*[tiab] OR suprahyoid*[tiab] OR mylohyoid*[tiab] OR geniohyoid*[tiab] OR "masticatory muscle"[tiab] OR "masticatory muscles"[tiab] OR "masticatory musculature"[tiab] OR "jaw muscle"[tiab] OR "jaw muscles"[tiab] OR "mandibular muscle"[tiab] OR "mandibular muscles"[tiab] OR "chewing muscle"[tiab] OR "chewing muscles"[tiab])	139
Scopus	TITLE-ABS-KEY ((brux* OR "sleep bruxism" OR "awake bruxism" OR "teeth grinding" OR "tooth grinding" OR "teeth clenching" OR "tooth clenching" OR "jaw clenching" OR "rhythmic masticatory muscle activity" OR "rhythmic masticatory muscle activities" OR RMMA) AND ("botulinum toxin" OR "botulinum toxins" OR "botulinum neurotoxin" OR "botulinum neurotoxins" OR "botulinum toxin type A" OR "botulinum neurotoxin type A" OR botox OR dysport OR xeomin OR bont OR "BoNT-A" OR bontA OR BTX OR "BTX-A" OR BTXA OR BTA OR onabotulinumtoxinA OR abobotulinumtoxinA OR incobotulinumtoxinA OR prabotulinumtoxinA OR letibotulinumtoxinA OR daxibotulinumtoxinA OR rimabotulinumtoxinB) AND (masseter* OR temporalis OR "temporalis muscle" OR "temporalis muscles" OR "temporal muscle" OR "temporal muscles" OR pterygoid* OR digastric* OR suprahyoid* OR mylohyoid* OR geniohyoid* OR "masticatory muscle" OR "masticatory muscles" OR "masticatory musculature" OR "jaw muscle" OR "jaw muscles" OR "mandibular muscle" OR "mandibular muscles" OR "chewing muscle" OR "chewing muscles")) AND LANGUAGE (english)	221
Central	#1 MeSH descriptor: [Bruxism] explode all trees; #2 (brux* OR “sleep bruxism” OR “awake bruxism” OR “teeth grinding” OR “tooth grinding” OR “teeth clenching” OR “tooth clenching” OR “jaw clenching” OR “rhythmic masticatory muscle activity” OR “rhythmic masticatory muscle activities” OR RMMA OR “mandibular bracing” OR “mandibular thrusting”):ti,ab,kw; #3 #1 OR #2; #4 MeSH descriptor: [Botulinum Toxins] explode all trees; #5 MeSH descriptor: [Botulinum Toxins, Type A] explode all trees; #6 (“botulinum toxin” OR “botulinum toxins” OR “botulinum neurotoxin” OR “botulinum neurotoxins” OR “botulinum toxin type A” OR “botulinum neurotoxin type A” OR “botulinum A toxin” OR “botulinum type A” OR “botulinum toxin type B” OR “botulinum neurotoxin type B” OR botox OR dysport OR xeomin OR myobloc OR neurobloc OR BoNT OR “BoNT-A” OR BoNTA OR BTX OR “BTX-A” OR BTXA OR BTA OR onabotulinumtoxinA OR abobotulinumtoxinA OR incobotulinumtoxinA OR prabotulinumtoxinA OR letibotulinumtoxinA OR daxibotulinumtoxinA OR rimabotulinumtoxinB):ti,ab,kw; #7 #4 OR #5 OR #6; #8 #3 AND #7.	74
WHO ICTRP	Advanced Search: Condition field: (bruxism OR "sleep bruxism" OR "awake bruxism"); Intervention field: (botulinum OR "botulinum toxin" OR "botulinum neurotoxin" OR Botox OR Dysport OR Xeomin OR onabotulinumtoxinA OR abobotulinumtoxinA OR incobotulinumtoxinA). The fields were combined using AND. Synonym searching was enabled. Recruitment status was set to All. No restrictions were applied by country, registration date, trial phase, participant age, or availability of results.	29

Note. CENTRAL, Cochrane Central Register of Controlled Trials; WHO ICTRP, World Health Organization International Clinical Trials Registry Platform.

Supplementary Table S3. Detailed numerical data for review-relevant efficacy and safety outcomes

Study and outcome	Results by group and assessment time	Comparative result
Alzaeem et al., 2025<br>Bruxism questionnaire (%)	Masseter + lateral pterygoid: baseline 75.00 +/- 33.07; 2 weeks 13.89 +/- 13.18; 6 weeks 16.67 +/- 12.50; 3 months 41.67 (SD NR); 6 months 52.78 +/- 23.20.<br>Masseter only: baseline 61.11 +/- 33.33; 2 weeks 13.89 (SD NR); 6 weeks 11.11 +/- 13.18; 3 months 36.11 +/- 25.35; 6 months 50.00 +/- 30.62.	Between-group p values: baseline 0.388; 2 weeks 1.000; 6 weeks 0.372; 3 months 0.624; 6 months 0.831.
Alzaeem et al., 2025<br>Masseter EMG (reported as mV)	Masseter + lateral pterygoid: right 643.72 +/- 119.21 to 295.77 +/- 134.44; left 649.04 +/- 166.32 to 272.10 +/- 98.40.<br>Masseter only: right 791.12 +/- 172.31 to 354.36 +/- 118.29; left 693.91 +/- 178.89 to 311.81 +/- 97.79.<br>Measurements: baseline to 2 weeks.	Both groups improved within group (all p<0.001). No significant between-group difference at 2 weeks was reported.
Alzaeem et al., 2025<br>Lateral pterygoid EMG (reported as mV)	Masseter + lateral pterygoid: right 679.96 +/- 131.80 to 250.79 +/- 74.71; left 732.64 +/- 219.61 to 231.92 +/- 54.94.<br>Masseter only: right 585.26 +/- 103.87 to 510.89 +/- 107.57; left 555.06 +/- 80.62 to 481.03 +/- 66.28.<br>Measurements: baseline to 2 weeks.	At 2 weeks, expanded treatment favored the lateral pterygoid EMG outcome on both sides (p<0.001). Baseline left-side EMG differed between groups (p=0.037).
Kim et al., 2024<br>Bite force (N)	Masseter only: baseline 674 +/- 414; 4 weeks 433 +/- 248; 8 weeks 406 +/- 286.<br>Masseter + anterior digastric: baseline 869 +/- 559; 4 weeks 682 +/- 466; 8 weeks 612 +/- 335.	Group-by-time interaction p=0.852; no significant incremental effect of anterior digastric injection.
Kim et al., 2024<br>Masseter thickness (mm)	Masseter only: baseline 14.24 +/- 1.81; 4 weeks 12.75 +/- 1.50; 8 weeks 13.04 +/- 1.71.<br>Masseter + anterior digastric: baseline 15.38 +/- 2.17; 4 weeks 13.88 +/- 2.28; 8 weeks 13.56 +/- 2.02.	Group-by-time interaction p=0.349.
Kim et al., 2024<br>Anterior digastric thickness and subjective findings	Anterior digastric thickness in expanded arm: baseline 6.0 +/- 2.0 mm; 4 weeks 5.9 +/- 1.5; 8 weeks 6.3 +/- 1.5. Self-reported decrease in sleep bruxism: 12/12 in each group. Side effects: 0/12 masseter-only; 2/12 (16.7%) expanded arm.	No significant reduction in anterior digastric thickness was reported. The two expanded-arm events were temporary difficulty closing the mouth and resolved by 8 weeks.
da Silva Ramalho et al., 2023<br>Pain VAS (0-10)	Masseter only: baseline 6.5 +/- 1.65; 15 d 1.9 +/- 1.792; 90 d 0.3 +/- 0.483; 120 d 0.5 +/- 0.707; 180 d 1.3 +/- 1.567.<br>Masseter + temporalis: baseline 7.6 +/- 1.84; 15 d 2.2 +/- 1.81; 90 d 0.9 +/- 1.73; 120 d 1.5 +/- 1.43; 180 d 2.5 +/- 2.27.	Between-group p values: baseline 0.125; 15 d 0.642; 90 d 0.815; 120 d 0.099; 180 d 0.349.
da Silva Ramalho et al., 2023<br>Treatment satisfaction VAS (0-10)	Masseter only: 15 d 9.00 +/- 1.00; 90 d 9.78 +/- 0.441; 120 d 9.56 +/- 0.726; 180 d 9.44 +/- 0.882.<br>Masseter + temporalis: 15 d 9.00 +/- 2.21; 90 d 9.20 +/- 1.62; 120 d 8.70 +/- 1.25; 180 d 8.40 +/- 1.26.	Between-group p values: 15 d 0.358; 90 d 0.597; 120 d 0.096; 180 d 0.066.
da Silva Ramalho et al., 2023<br>Posterior bite force (N)	Masseter only: baseline 339 +/- 190.4; 15 d 174 +/- 61.3; 90 d 207 +/- 84.6; 120 d 265 +/- 116.4; 180 d 320 +/- 167.8.<br>Masseter + temporalis: baseline 381 +/- 256; 15 d 192 +/- 115; 90 d 238 +/- 129; 120 d 283 +/- 139; 180 d 337 +/- 182.	Between-group p values: baseline 0.796; 15 d 0.971; 90 d 0.853; 120 d 1.000; 180 d 0.971.
Cruse et al., 2022<br>Bruxism Index (events/hour)	All analyzed participants (n=22): baseline 8.29 +/- 2.88. Active phase: 4 weeks 6.67 +/- 4.09; 12 weeks 7.38 +/- 3.55. Placebo phase: 4 weeks 8.33 +/- 4.11; 12 weeks 7.67 +/- 3.50.	Active versus placebo: mean difference -1.66 at 4 weeks (p=0.003); no difference at 12 weeks (p=0.58). Among injection groups, the three-muscle arm had change -2.15 versus placebo (p=0.009); masseter-only p=0.287; two-muscle p=0.073; overall group difference p=0.15.
Cruse et al., 2022<br>Patient-reported outcomes	HIT-6: baseline 60.8 +/- 8.77; active 4/12 weeks 59.2 +/- 8.56 / 57.9 +/- 8.64; placebo 4/12 weeks 55.8 +/- 9.49 / 58.1 +/- 9.22.<br>Pain Rating Index: 16.2 +/- 10.6; 12.6 +/- 8.8 / 14.3 +/- 12.5; 13.1 +/- 10.6 / 14.8 +/- 10.1.<br>Pain VAS: 56.9 +/- 26.7; 41.9 +/- 26.6 / 44.6 +/- 27.1; 46.9 +/- 28.8 / 53.9 +/- 29.9.<br>Short-Form McGill overall intensity: 2.93 +/- 1.28; 2.25 +/- 1.11 / 2.12 +/- 1.24; 2.48 +/- 1.39 / 2.58 +/- 1.33.<br>Epworth Sleepiness Scale: 7.55 +/- 4.82; 6.65 +/- 4.61 / 6.85 +/- 4.53; 7.15 +/- 4.83 / 7.42 +/- 4.80.	No significant active-versus-placebo difference: HIT-6 p=0.65; Pain Rating Index p=0.81; pain VAS p=0.33; McGill overall intensity p=0.19; Epworth p=0.92.
Cruse et al., 2022<br>Adverse events	Transient chewing weakness/fatigue: 4 participants (masseter-only 1; masseter+temporalis 1; three-muscle 2). Lower facial weakness: 1 participant in two-muscle arm, lasting 10 days. Mild bruising: 1 masseter-only participant. Injection discomfort led to withdrawal: 1 three-muscle participant.	No serious treatment-related event was reported in this trial.
Shim et al., 2014<br>RMMA episode variables at 4 weeks	RMMA index (/h): masseter-only 3.27 +/- 1.41 to 2.82 +/- 2.47; masseter+temporalis 2.81 +/- 1.38 to 2.88 +/- 2.59.<br>Bursts/h, median (range): 10.10 (6.27-23.52) to 6.82 (0.51-59.06); 11.96 (2.54-50.45) to 12.45 (0.00-	Intergroup p values: RMMA index 0.573; bursts/h 0.997; bursts/episode 0.457; episode duration 0.339. No significant incremental effect on episode occurrence.

	47.12).<br>Bursts/episode: 4.07 +/- 1.11 to 4.69 +/- 2.80; 5.02 +/- 1.78 to 4.72 +/- 3.05.<br>Episode duration: 5.67 +/- 1.22 to 5.99 +/- 2.99; 6.82 +/- 1.37 to 5.79 +/- 3.31.	
Shim et al., 2014<br>Peak EMG amplitude, median (range), microvolts	MVC masseter: masseter-only 216.30 (39.00-800.50) to 43.10 (26.70-130.10); expanded 223.15 (81.20-333.80) to 78.00 (34.20-169.10).<br>MVC temporalis: 169.72 (105.50-515.50) to 171.05 (64.70-414.00); expanded 226.35 (104.90-432.90) to 104.45 (40.20-218.20).<br>RMMA masseter: 290.47 (31.12-741.62) to 72.41 (29.41-141.58); expanded 215.71 (114.32-390.86) to 72.30 (32.87-98.90).<br>RMMA temporalis: 277.63 (87.42-553.53) to 194.80 (45.00-383.95); expanded 306.41 (160.99-377.71) to 83.00 (17.24-283.44).	Intergroup p values: MVC masseter 0.185; MVC temporalis 0.010; RMMA masseter 0.916; RMMA temporalis 0.025. The additional temporalis injection produced a temporalis-specific EMG effect.
Shim et al., 2014<br>Subjective symptoms and tolerability	Reported decreased tooth grinding: 4/10 masseter-only and 5/10 expanded arm. Morning jaw stiffness reduction: 47.50% +/- 15.86% versus 57.50% +/- 30.30%. Perceived masticatory-force reduction: 30.00% +/- 17.64% versus 40.50% +/- 18.33%. Fourteen of 20 reported discomfort from reduced force; 3 reported masticatory difficulty.	Between-group p values: tooth grinding 1.000; morning stiffness 0.371; perceived force reduction 0.208.

Note. Values are mean +/- standard deviation unless otherwise stated. This table prioritizes outcomes relevant to comparative muscle targeting and does not reproduce ancillary sleep-architecture variables. Within-group improvement should not be interpreted as evidence of incremental benefit from adding another muscle. Reconstructed values are identified in the source/notes column. Alzaeem et al. report EMG values in 'mV' in the article; that unit is preserved here without conversion, although the reported unit may warrant clarification from the authors.

Supplementary Table S4. Linked reports and trial registrations for the included studies

Underlying study	No. of linked reports	Primary full-text report	Companion report(s)	Original trial registration	Use in the review
Alzaeem et al., 2025	2	Alzaeem MN, Karkoutly M, Haddad I, Brad B. J Stomatol Oral Maxillofac Surg. 2025;126:102404. DOI: 10.1016/j.jormas.2025.102404.	No separate conference abstract identified among the retained reports.	ISRCTN13377883. Registered 20 Dec 2024. The article reports study conduct from Jan to Nov 2024.	Primary source for participant, injection, and efficacy data; registry used to verify design, prespecified outcomes, and registration timing.
Kim et al., 2024	3	Kim SB, Choi YJ, Kim ST, Kim HJ. J Oral Rehabil. 2024;51:2125-2132. DOI: 10.1111/joor.13800.	Kim S. Same title. Journal of Sleep Research. 2024;33. Conference poster abstract indexed in CENTRAL via Embase.	KCT0008721, Clinical Research Information Service (Republic of Korea).	Full article used for extraction; poster and registry linked to verify that they describe the same 24-participant trial and to supplement registration/intervention details.
da Silva Ramalho et al., 2023	2	da Silva Ramalho JA, Palma LF, Ramalho KM, Tedesco TK, Morimoto S. Saudi Dent J. 2023;35:53-60. DOI: 10.1016/j.sdentj.2022.12.008.	No separate conference abstract identified among the retained reports.	ReBEC: RBR-39ght5; WHO Universal Trial Number: U1111-1235-5386. The full article reports the WHO UTN and identifies ReBEC; the ReBEC identifier was confirmed from the linked registry record.	Full article used for extraction; registry used to verify trial identity, design, and prespecified outcomes.
Cruse et al., 2022	2	Cruse B, Dharmadasa T, White E, et al. BMJ Neurol Open. 2022;4:e000328. DOI: 10.1136/bmjno-2022-000328.	No separate conference abstract retained for this study.	ACTRN12618001430224, Australian New Zealand Clinical Trials Registry.	Full article used for extraction; registry used to verify crossover design, intervention groups, outcomes, and registration timing.
Shim et al., 2014	2	Shim YJ, Lee MK, Kato T, Park HU, Heo K, Kim ST. J Clin Sleep Med. 2014;10:291-298. DOI: 10.5664/jcsm.3532.	No separate conference abstract identified among the retained reports.	NCT01336439, ClinicalTrials.gov.	Full article used for extraction; registry used to verify intervention groups, outcomes, and registration timing.

Note. Companion reports and registry records were linked to the corresponding underlying study and were not counted as additional studies or participants. Clinical outcome synthesis was based primarily on the five full-text journal articles. Registry-only details are identified as originating from the linked registry record rather than from the article text. In total, the five unique studies were represented by 11 reports: five primary journal articles, five trial registry records, and one conference abstract.

Supplementary Table S5. Final data-charting form used for the scoping review

Domain	Data item
Study identification	Author, year, country, setting, study design
Participants	Sample size, age, sex, bruxism phenotype, diagnostic/assessment method, relevant comorbidities
Intervention	Botulinum toxin preparation, muscles injected, dose per muscle, total dose, number of injection points, dilution/concentration, guidance method
Comparator	Muscles injected, dose per muscle, total dose, injection technique
Outcomes	Outcome domain, measurement instrument, assessment time points
Effectiveness data	Group-specific results, between-group comparisons, effect estimates/p values where reported
Safety	Adverse events, withdrawals, tolerability
Trial information	Registration identifier, registration timing, companion reports
Data provenance	Directly reported value, reviewer-calculated value, or review-level interpretation
Notes	Missing, unclear, or internally inconsistent information

Supplementary Table S6. Bruxism phenotype, assessment approach, and comparative findings of the included studies

Study	Bruxism phenotype	Assessment approach	Diagnostic/assessment basis	Main comparative finding
Alzaeem et al., 2025	Unspecified	Instrumental and non-instrumental	Bruxism questionnaire ≥50%, increased masseter and lateral pterygoid EMG activity, and clinical dental erosion	No between-group difference in questionnaire scores or masseter EMG; lateral pterygoid EMG favored the expanded strategy
Kim et al., 2024	Sleep bruxism	Non-instrumental	History of clenching during sleep ≥3 times/week, morning jaw stiffness, and clinical tooth wear	No significant between-group difference in bite force or masseter thickness; no reduction in anterior digastric thickness
da Silva Ramalho et al., 2023	Sleep or awake bruxism	Non-instrumental	Self-reported sleep or awake bruxism for ≥6 months plus bilateral myofascial pain	No significant between-group differences in pain, treatment satisfaction, or bite force
Cruse et al., 2022	Sleep bruxism	Instrumental	ICSD-R criteria plus baseline Bruxism Index >5 based on three nights of surface EMG	No statistically significant difference among the three active injection strategies
Shim et al., 2014	Sleep bruxism	Instrumental	Clinical sleep-bruxism criteria with baseline and follow-up video-polysomnography; participants without baseline RMMA were excluded from analysis	No between-group difference in RMMA episode occurrence; additional temporalis injection produced greater temporalis-specific EMG reduction

Note. EMG, electromyography; ICSD-R, International Classification of Sleep Disorders-Revised; RMMA, rhythmic masticatory muscle activity. Instrumental assessment refers to electromyographic or polysomnographic assessment; non-instrumental assessment refers to self-report, questionnaire, and/or clinical examination.

Supplementary Table S7. Prespecified classification rules for comparative botulinum toxin injection strategies

Classification category	Operational rule	Interpretation in the synthesis	Included studies
Direct add-on comparison	The same masseter dose was used in both groups, with one or more additional muscles injected in the expanded-treatment group.	Considered directly informative for anatomical add-on targeting when the same toxin formulation and masseter dose were used. If adding another muscle increased the total toxin dose, the comparison was considered dose-unmatched.	Alzaeem et al., 2025; Kim et al., 2024; da Silva Ramalho et al., 2023; Shim et al., 2014
Dose-escalating multi-muscle comparison	The number of muscles targeted and the total toxin dose increased across multiple active injection strategies.	Anatomical targeting and dose escalation could not be separated.	Cruse et al., 2022
Alternative non-masseter strategy	A masseter-only regimen was compared with a regimen targeting different muscle(s) without a shared masseter injection pattern.	Represents an alternative anatomical strategy rather than an add-on comparison.	None identified
Other comparative strategy	Both groups received botulinum toxin but differed in muscle-targeting pattern and did not meet the preceding definitions.	Retained to capture other eligible comparative injection patterns.	None identified

Note. Unrepresented categories were retained to reflect the prespecified review scope. Comparisons with higher total doses in expanded arms were considered dose-unmatched. Toxin doses were not compared across formulations because potency units are product-specific.
